# Hamstring injury rates have increased during recent seasons and now constitute 24% of all injuries in men’s professional football: the UEFA Elite Club Injury Study from 2001/02 to 2021/22

**DOI:** 10.1136/bjsports-2021-105407

**Published:** 2022-12-06

**Authors:** Jan Ekstrand, Håkan Bengtsson, Markus Waldén, Michael Davison, Karim M Khan, Martin Hägglund

**Affiliations:** 1 Department of Health, Medicine and Caring Sciences, Linköping University, Linköping, Sweden; 2 FIFA Medical Centre of Excellence, London, Isokinetic Medical Group, London, UK; 3 Family Practice & Kinesiology, The University of British Columbia, Vancouver, British Columbia, Canada

**Keywords:** Injuries, Athletic Performance, Epidemiology, Hamstring Muscles, Soccer

## Abstract

**Objectives:**

To: (1) describe hamstring injury incidence and burden in male professional football players over 21 seasons (2001/02 to 2021/22); (2) analyse the time-trends of hamstring muscle injuries over the most recent eight seasons (2014/15 to 2021/22); and (3) describe hamstring injury location, mechanism and recurrence rate.

**Methods:**

3909 players from 54 teams (in 20 European countries) from 2001/02 to 2021/22 (21 consecutive seasons) were included. Team medical staff recorded individual player exposure and time-loss injuries. Time-trend analyses were performed with Poisson regression using generalised linear models.

**Results:**

2636 hamstring injuries represented 19% of all reported injuries, with the proportion of all injuries increasing from 12% during the first season to 24% in the most recent season. During that same period, the percentage of all injury absence days caused by hamstring injuries increased from 10% to 20%. Between 2014/15 and 2021/22, training hamstring injury incidence increased (6.7% annually, 95% CI 1.7% to 12.5%) as did burden (9.0% annually, 95% CI 1.2% to 18.3%). During those years, the match hamstring injury incidence also increased (3.9% annually, 95% CI 0.1% to 7.9%) and with the same trend (not statistically significant) for match hamstring injury burden (6.2% annually, 95% CI −0.5% to 15.0%).

**Conclusions:**

Hamstring injury proportions—in number of injuries and total absence days—doubled during the 21-year period of study. During the last eight seasons, hamstring injury rates have increased both in training and match play.

**WHAT IS ALREADY KNOWN ON THIS TOPIC**

- Hamstring muscle injuries increased in incidence in men’s professional football from 2001 to 2014.
- Since then, players train more intensely and their match calendar is more crowded. Many professional teams aim to prevent muscle injury as part of their strength and conditioning programmes.

**WHAT THIS STUDY ADDS**

- During the recent eight seasons (2014/15 to 2021/22), the incidence and burden of hamstring injuries during training and match play have increased significantly.
- The proportion of injuries diagnosed as hamstring injuries increased from 12% in 2001/02 to 24% in 2021/22.
- The proportion of all injury absence days caused by hamstring injuries has increased from 10% in 2001/02 to 20% in 2021/22.
- Around 18% of all reported hamstring injuries were recurrences with over two-thirds occurring within 2 months of the footballer’s return to play.

**HOW THIS STUDY MIGHT AFFECT RESEARCH, PRACTICE OR POLICY**

- These data on incidence and burden of hamstring injuries provide a strong rationale for teams to keep focusing on preventing first and recurrent hamstring injuries.
- The high rate of recurrent hamstring injury within 2 months of return to play suggests this period is a particularly important time for team clinicians to: (1) carefully monitor players completing evidence-based rehabilitation; (2) manage training and match loads; and (3) maintain preventive programmes.

## Introduction

In 1999, the Union of European Football Associations (UEFA) conceived a research project—the Elite Club Injury Study (ECIS)—which had the aim of evaluating the risk of injury for top-level men’s football players in Europe. Its ultimate purpose was to reduce football injuries and increase player safety.^1–3^ We have previously reported that hamstring injuries constituted 12–17% of all time-loss injuries in male professional football,^2 4^ and that hamstring injury was the most common recurrent injury in football players.^5^

The incidence of match-related hamstring injuries was stable from 2001/02 to 2013/14, even though the incidence of training-related hamstring injuries increased by an average of 4% annually during that period.^6^ Researchers have proposed various interventions to stem the tide of hamstring injuries, and teams have variably embraced systematic hamstring prevention programmes.^7^ Whether men’s professional football clubs have flattened the curve of hamstring injuries since 2014 is unknown. If hamstring injury incidence and burden have risen in recent years, it might imply that current preventative measures are not working. On the other hand, if hamstring injury incidence and burden have decreased, it could be due to effective prevention strategies. We know of no comparable surveillance studies of hamstring injuries in any professional sport (not just men’s football). Additionally, little is known about whether hamstring injuries occur more commonly in the biceps femoris or the semimembranosus/semitendinosus muscles,^8^ and about the proportion of hamstring injuries that meet the criteria for structural (muscle fibre) injury.^9^

The primary objective of this ECIS study was to describe the hamstring injury incidence and burden in professional men’s football over 21 seasons (2001/02 to 2021/22). Our secondary aim was to analyse the time trends of hamstring muscle injuries over the most recent eight seasons (2013/14 to 2021/22) to see if previously reported trends have continued. Further objectives were to compare the prevalence of (1) biceps femoris injuries and semitendinosus/semimembranosus injuries, and (2) structural and functional hamstring injuries.

## Methods

The ECIS data collection began in July 2001,^10^ and the study recently completed its 21st season (2021/22). The data in this prospective hamstring sub-study cover the period from 2001/02 (inception) to 2021/22 (21 consecutive seasons). Each year, all teams that qualify for the UEFA Champions League (UCL) group stage are invited by UEFA to participate in ECIS. Teams that have been enrolled for at least one season may continue in ECIS even if they do not qualify for the UCL group stage during a subsequent season.

### Study population

*Inclusion criteria at team-level*: to increase homogeneity in this hamstring sub-study it was decided to include data from ECIS teams only during seasons that they participated in the UCL group stage.^11^ We did this for two reasons:

1. there could be some important differences between teams participating in the UCL group stage and teams that did not (eg, fixture congestion, tactics, playing style, financial conditions, etc)
2. the number of teams that failed to qualify for the UCL group stage following their initial inclusion in ECIS has increased during the course of the study.

During the 21 seasons, 54 teams from 20 European countries (total 323 team-seasons) met this criteria and were included for analysis.

*Inclusion criteria at player-level*: all players with a first team contract within the included teams were invited to volunteer for the study. A total of 3909 players (total 9728 player-seasons) who gave their written informed consent were included. Players who left or joined the team during the season (eg, due to club transfer) were included during their time on the team. No player refused to participate in the study.

### Study procedure

Included teams assigned a contact person (a member of the medical staff) who was responsible for registering data. The contact person was given a manual that provided the methodology and operational definitions used in the study (table 1). These definitions have been constant throughout the entire period of ECIS. New variables were added for the 2011/12 and subsequent seasons, describing affected muscles (biceps femoris or semitendinosus/semimembranosus) and injury classification according to the Munich muscle injury classification system (functional or structural).^9^ Functional muscle disorders was the term for disorders without macroscopic evidence of fibre tear on MRI; structural muscle injuries refer to cases where there was macroscopic evidence of fibre tear on MRI. Sub-classifications were presented for each type.^9^

**Table 1:** Operational definitions

Training session	Team training that involved physical activity under the supervision of the coaching staff
Match	Competitive or friendly match against another team
Injury	Any physical complaint sustained by a player that resulted from a football match or football training and led to the player being unable to take full part in future football training or match play.
Hamstring injury	A traumatic distraction or gradual onset injury to the hamstring muscle group
Functional muscle injury	Acute indirect muscle disorder without macroscopic evidence of muscle tear
Structural muscle injury	Acute indirect muscle injury with macroscopic evidence of muscle tear
Injury severity	The number of lay-off days caused by the injury between the injury date and the date when the medical team declared the player as ready for full participation in team training and availability for match play
Recurrent injury	Injury of the same type and at the same site as an index injury occurring previously during the same season
Early recurrence	Recurrent injury that occurs within 2 months after return to full participation from the index injury
Injury incidence	Number of injuries per 1000 player hours ((Σ injuries/Σ exposure hours)×1000)
Injury burden	Number of lay-off days per 1000 player hours ((Σ lay-off days/Σ exposure hours)×1000)

The study was carried out by the Football Research Group (FRG) on behalf of UEFA. FRG is an international research team conducting studies on football injuries. Teams were asked to provide the study group with exposure and injury data each month. All data were reviewed by one FRG researcher in the beginning, but lately by two FRG researchers (as the study has expanded), who performed quality control (ensuring that the data were complete and complied with the study protocol). These controllers had been trained in football epidemiology and held either a PhD (HB, MW, MH, JE, Matilda Lundblad and Karolina Kristenson) or a Master’s degree (Anna Hallén) in this field. If any missing or unclear data were identified during this review process, immediate feedback was sent by the quality controllers to the club’s contact person to complete or correct the data. Further, a statistician (Henrik Hedevik) scrutinised the database after each season, seeking to flush out other possible data input errors. The study methods were consistent with the prevailing international consensus statements on football injury epidemiology; detailed descriptions of the methods,^12–14^ and the FRG study group,^15^ have been published previously.

### Exposure

Individual football exposure was registered in minutes on a standard attendance record. This form included information about the duration for each exposure (including training and match play), or whether players were absent due to injury, illness, national team duty, or other reasons.

### Injury

Injury was defined according to time-loss (table 1).^12^ For each injury, the contact person was asked to complete an injury card including a free text diagnosis. Based on the information on the injury card, members of the study group classified the injury using the Orchard Sports Injury Classification System (OSICS).^16 17^ OSICS version 2 was used throughout the entire study period while OSICS version 10 was added from the 2011/2012 season. Since the 2011/12 season, injuries were also described using the Munich muscle injury classification,^9 18^ which distinguishes between functional and structural muscle injuries. We have not changed any element of data collection that might bias how we measure injury incidence.

Injury severity was defined by the number of lay-off days that passed between the injury occurrence and return to play, and categorised in five different categories: slight (0 days), minimal (1–3 days), mild (4–7 days), moderate (8–28 days), severe (>28 days).^1 2 4^

Injuries that did not occur during a specific identifiable event (eg, overuse/gradual onset injuries) were assigned to the last activity (training or match) that the injured player completed before being removed from full participation in team activities.

For match injuries the injury card also contained information about the match minute in which the injury occurred.

### Patient and public involvement

Before the launch of the first season of ECIS, the medical staff of the participating teams (as per May 2001) were invited to comment on the study procedures and definitions.^12^ This research was done without patient (player) involvement; patients (players) were not invited to comment on the study design or to contribute to the drafting of this document. We propose to include players and health professionals in the knowledge translation that will follow the publication of this study.

### Equity, diversity, and inclusion

This study focused exclusively on male professional football players. We acknowledge that our author list does not reflect the diversity of the sport and exercise medicine/rehabilitation community. A women’s ECIS (WECIS) was launched in July 2017 in collaboration with UEFA with similar data collection on hamstring injuries. The WECIS is led by Anna Hallén and the author team includes two female football doctors.

### Data analyses

Time-trend analyses were performed with Poisson regression using generalised linear models (GENLIN) with injury count data (number of injuries and number of lay-off days) as the dependent variables, season as covariate, and with natural log of the exposure variable (total hours, training hours, and match hours) as offset variable. We used the model-based estimator for the covariance matrix, and Wald statistics to calculate the p value and 95% CI. Time-trends in injury incidence and injury burden are expressed as the annual % change between seasons with a 95% CI. Time-trend analyses were performed (1) for the entire 21-season study period, and (2) for the sub-period 2014/15 to 2021/22 (since the 2001/02 to 2013/14 seasons have been covered in a previous report).^6^

Injury incidence was defined as the number of injuries per 1000 hours and described with 95% CI. To compare the injury incidence between training and match play, a rate ratio (RR) with a corresponding 95% CI was calculated and tested for statistical significance using Z-statistics. Injury burden was calculated for all participating teams in each of the 21 seasons separately and are presented as the mean injury burden for these seasons with corresponding SD. Injury severity was expressed as the median number of lay-off days with the corresponding IQR. The number of lay-off days was compared between structural and functional injuries, as well as between biceps femoris injuries and semitendinosus/semimembranosus injuries, using the Mann-Whitney U test due to skewed data distribution.

The proportion of injuries representing the different severity categories, injury mechanisms, period of match, and recurrences were compared between structural and functional injuries, and between biceps femoris injuries and semitendinosus/semimembranosus (both available from 2011/12), using the χ^2^ test. The Z-test was used for pairwise comparisons with Bonferroni correction for multiple comparisons. A one-sample proportional Z-test was used to compare the proportion of injuries occurring in different 15 min periods of match halves. Analyses were two-sided and the significance level was set at p<0.05. All analyses were conducted in Statistical Package for the Social Sciences (SPSS) v28.

## Results

### Time trends during the 21 seasons

During the 2001/02 to 2021/22 seasons, team medical staff reported 2636 hamstring injuries during 2 131 561 total exposure hours; 922 injuries (34%) during 1 787 823 training hours and 1714 injuries (66%) during 343 738 match hours. The proportion of reported injuries that were diagnosed as hamstring injuries increased from 12% in the first season to 24% in the last season and constituted 19% of all 14 057 injuries registered during the 21-season study period. In terms of absence days, hamstring injuries caused 14% of the total injury lay-off days, increasing from 10% to 20% between the 2001/02 and the 2021/22 seasons. The hamstring injury incidence was 10 times higher during match play than training (4.99/1000 hours vs 0.52/1000 hours; RR 9.67, 95% CI 8.93 to 10.47). The median lay-off following a hamstring injury was 13 days (IQR 7–22). In general, 20% of players missed training or match play due to hamstring injury during a season, and a 25-player squad can expect about eight hamstring injuries per season.

Of all the hamstring injuries, 475 (18%) were recurrences, and early recurrences (within 2 months, n=325) made up 69% of these. Recurrences were nine times more likely to occur in matches than in training (0.88 vs 0.10; RR 9.25, 95% CI 7.67 to 11.15) as were early recurrences (0.61 vs 0.07; RR 9.25, 95% CI 7.37 to 11.60).

**Figure 1:**
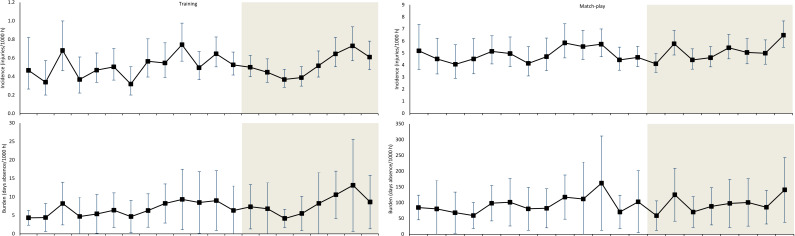
Development of hamstring injury incidence and injury burden over the study period. Injury incidence is defined as the number of injuries per 1000 hours of exposure, presented with 95% CI. Injury burden is defined as the number of absence days caused per 1000 hours of exposure and is presented as the mean of participating teams with SD. The shaded area represents the most recent eight-season period which has not been previously published.

**Figure 2:**
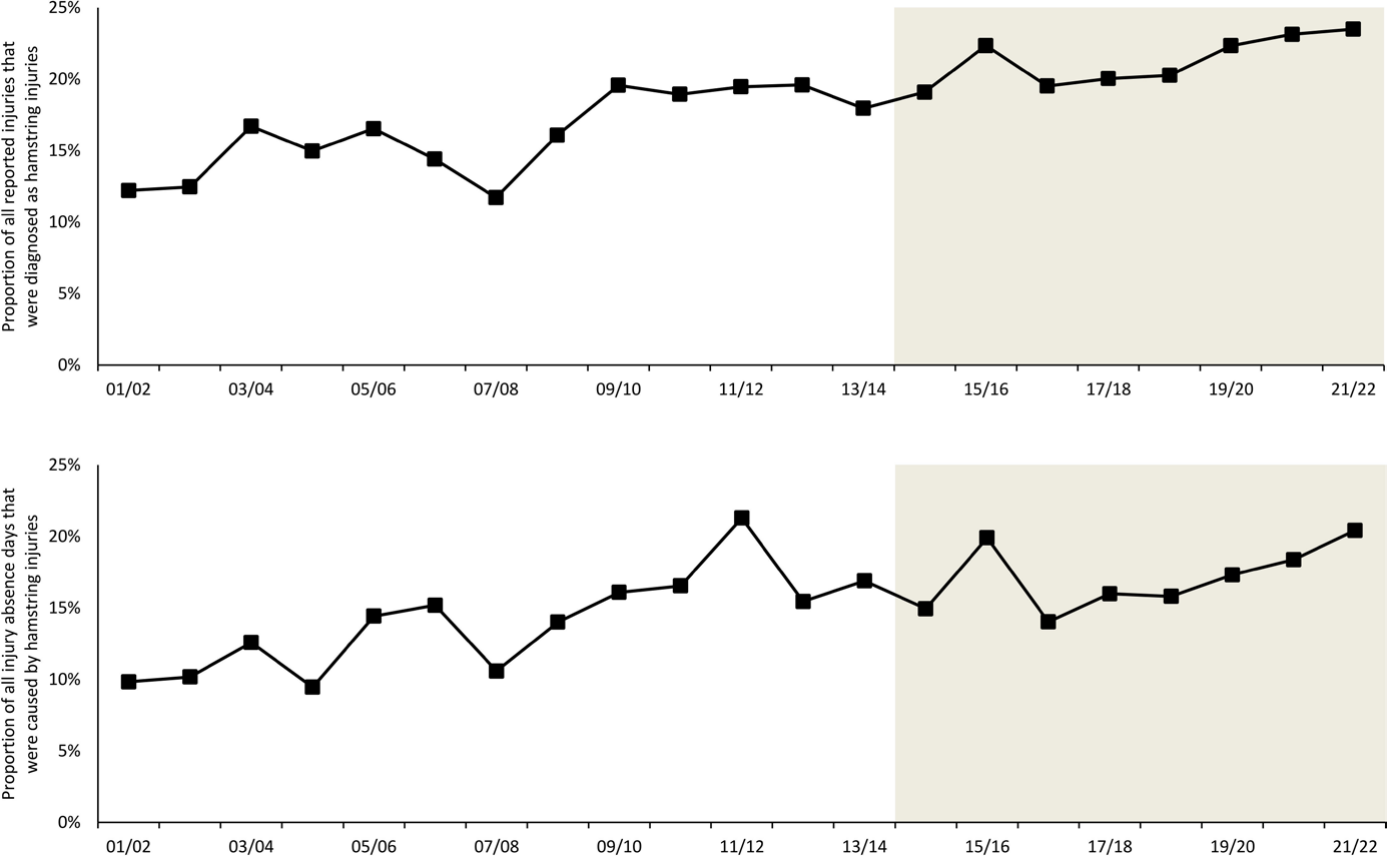
Development of the proportion of all reported injuries that were diagnosed as hamstring injuries and the proportion of all injury absence days caused by hamstring injuries over the study period. The shaded area represents the most recent eight-season period which has not been previously published.

Time-trend analyses over all 21 seasons showed no significant trends for overall football injury incidence (0.7% annually, 95% CI −0.6% to 1.9%) or injury burden (1.4% annually, 95% CI −0.1% to 3.0%) (figures 1 and 2).

### Time-trends during 2014/15-2021/22

In the period 2014/15 to 2021/22, time trend analysis revealed a significant increase in both training hamstring injury incidence and training hamstring injury burden. During the same period, the match hamstring injury incidence also increased with the same trend (not statistically significant) observed for the match hamstring injury burden (table 2).

**Table 2:** Results from the Poisson regression analyses showing the seasonal change in hamstring injury incidence between the 2014/15 and 2021/22 seasons

	% Annual change	95% CI	P value
Training			
Hamstring injury incidence	6.7%	(1.7 to 12.5)	0.009
Hamstring injury burden	9.0%	(1.2 to 18.3)	0.024
Match play			
Hamstring injury incidence	3.9%	(0.1 to 7.9)	0.045
Hamstring injury burden	6.2%	(−0.5 to 15.0)	0.116

Results are expressed as the annual % change with 95% CI. Positive values represent an increasing trend and negative values a decreasing trend.

### Characterisation by injury types during 2001/02 to 2021/22

Injury characteristics of structural/functional and lateral/medial injuries are presented in table 3. Between the 2011/12 and 2021/22 seasons, 1819 hamstring injuries were reported and classified according to the Munich muscle injury classification system (no classification available for 24 injuries). The majority of these (n=1312, 71%) were classified by the team doctors as structural injuries. Structural injuries were associated with longer lay-off than functional injuries (median absence 17 vs 6 days) (table 3). Running/sprinting was the most common injury mechanism (62% of structural injuries and 51% of functional injuries). The χ^2^ test revealed a significant overall difference in the frequency of injury mechanisms between structural and functional injuries (p<0.001).

**Table 3:** Hamstring injury characteristics

	All injuries	Injury type		Injury location	
		Structural	Functional	Biceps femoris	Semimembranosus/ semitendinosus
Injury lay-off					
Lay-off days, median (IQR)	13 (7 to 22)	17 (11 to 25)*	6 (4 to 10)*	16 (10 to 25)*	15 (8 to 23)*
Injury severity					
Slight, n (%)	9 (0%)	2 (0%)	1 (0%)	2 (0%)	0 (0%)
Minimal, n (%)	222 (8%)	32 (2%)*	102 (20%)*	27 (3%)*	19 (7%)*
Mild, n (%)	508 (19%)	128 (10%)*	205 (40%)*	122 (12%)*	43 (16%)*
Moderate, n (%)	1514 (57%)	886 (68%)*	187 (37%)*	709 (67%)	164 (62%)
Severe, n (%)	383 (15%)	264 (20%)*	12 (2%)*	194 (18%)	39 (15%)
Total, n	2636	1312	507	1054	265
Injury mechanism					
Running/sprinting, n (%)	1230 (61%)	790 (62%)*	232 (51%)*	654 (64%)*	140 (56%)*
Stretching, n (%)	103 (5%)	82 (6%)*	14 (3%)*	52 (5%)*	24 (10%)*
Overuse/gradual onset, n (%)	249 (12%)	60 (5%)*	150 (33%)*	63 (6%)	23 (9%)
Other, n (%)	449 (22%)	334 (26%)*	55 (12%)*	253 (25%)	63 (25%)
Total, n	2031	1266	451	1022	250
Recurrences					
Recurrences, n (%)	477 (18%)	234 (18%)	83 (16%)	195 (19%)	45 (17%)
Early recurrences, n (%)	325 (12%)	163 (12%)	47 (9%)	136 (13%)	29 (11%)
Total, n	2636	1312	507	1054	265

Injury mechanisms were registered starting from the 2008/09 season and are thus missing for 605 injuries.

*Significant difference in lay-off days, injury severity proportion, injury mechanism proportion, or recurrence proportion between injury types or injury locations.

**Figure 3:**
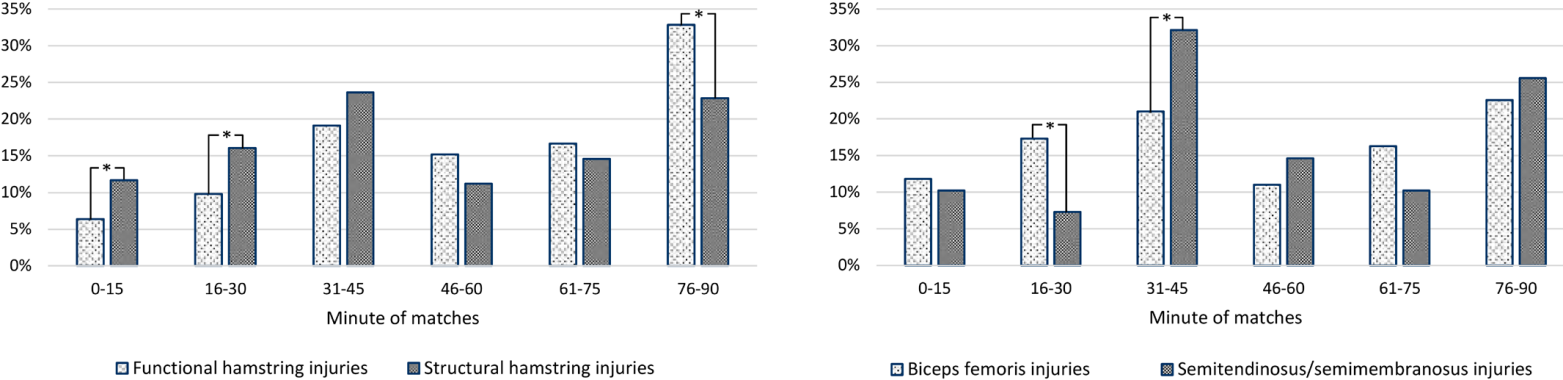
Distribution of different hamstring injury types and locations in different periods of matches. Asterisks indicate significantly different proportions of biceps femoris to semimembranosus/semitendinosus or functional to structural injuries.

Almost 50% of match hamstring injuries occurred during the last 15 min of the first and second halves, which deviated from the expected match distribution (p<0.001) (figure 3). A significant difference (p=0.002) in the frequency of structural and functional injuries in different match periods was observed (figure 3).

### Characterisation by injury location

Of the 1843 injuries reported since the 2011/12 season, 1319 (72%) were given diagnoses specific to either biceps femoris or semitendinosus/semimembranosus. Biceps femoris injuries (n=1054, 80%) were more common, especially in match play (n=761, 84%).

The χ^2^ test revealed a significant overall difference in the frequency of injury mechanisms between biceps femoris and semitendinosus/semimembranosus injuries (p=0.009), with a higher proportion of lateral injuries caused by running/sprinting while fewer lateral injuries were caused by stretching. Lateral injuries were also associated with longer absence than medial injuries (p=0.010) with a larger proportion of the medial injuries being minimal or mild (p=0.009).

A significant difference in the frequency of biceps femoris and semimembranosus/semitendinosus injuries in different match periods was observed (p<0.001) (figure 3).

## Discussion

This unique long-term ECIS dataset allows us to explore 21-year trends for hamstring injuries in men’s professional football in Europe.

- The most *important* findings were that the proportion of all injuries diagnosed as hamstring injuries increased from 12% to 24%, and that the proportion of all injury absence days caused by hamstring injuries increased from 10% to 20% during the 21-year study period.
- The most *worrying* finding was that the injury rates have increased during the recent eight seasons.
- The most *surprising* finding was that structural injuries were more common than functional injuries during the most recent 11 seasons.
- The most *expected* findings (extending previous studies) were that hamstring injuries were: (1) most commonly due to running/sprinting; (2) more likely to occur in the last 15 min of match halves; (3) affect the biceps femoris rather than the semimembranosus/semitendinosus muscles; and (4) predisposed to recur within 2 months in the same location.

### Can we explain why hamstring injuries are contributing an increasing proportion of all injuries?

Over the past 21 years, in professional men’s football, hamstring injuries have increased substantially as a proportion of the total number of reported injuries and as a proportion of injury burden. This is due to a combination of a decrease in other injuries (such as ligament injuries)^2 11^ and an absolute increase in the number of hamstring injuries per unit of exposure. Before we speculate as to why hamstring injuries have increased, we provide three reasons to underscore that we feel our data are valid—they are not the result of a secular trend in reporting (a bias). We note that: (1) we have not changed our diagnostic criteria; (2) the incidence data are based on time loss (not a clinician’s impression); and (3) there has been no increase in ‘all injuries’ within our study. The latter would be the case if the clinicians who collected data had become more attuned or motivated to capture injuries.

### Can we explain why hamstring injury rates are still high and even increasing during recent seasons?

This is a prospective epidemiological study revealing significant associations. Causative factors cannot be evaluated using this study design; we do not know the reasons for the observations.^11^ However, after 21 years of monthly contacts with these Champions League teams, we respectfully propose two hypotheses.

First, the intensity of elite men’s football has increased over at least a period of the years that are included in the current study.^19^ Current football practice includes a large volume of high intensity football actions.^20 21^ Professional players now undertake more high-intensity activities per match than they did previously and they also run faster than their predecessors.^19^ In our study, 61% of hamstring injuries occurred while the player was running/sprinting. We postulate that the number of hamstring injuries increased over time due to a greater number of high-risk activities in later years.

Compounding the pressure on hamstrings associated with football intensity is the increase in the total amount of international team travel and matches. This is often referred to as the problem of the crowded player calendar.^22^ Professional players now work year-round apart from a 4–6 week break between seasons.^22^ Even during the traditional break between seasons, players are often required to undertake pre-season tours which require intercontinental travel.^23^ This relentless travel and playing demand is associated with players being limited to fewer training sessions during the pre-season period^23^; training sessions may lower injury risk.^23^

### Why are the hamstring injury rates not decreasing despite studies showing that the Nordic Hamstring Exercise can reduce injury rates?

The Nordic Hamstring Exercise programme has been promoted for injury prevention,^24^ and may reduce hamstring injuries by 65–70%.^25 26^ However, the programme has not been widely adopted in men’s professional football in Europe.^24 27^ Challenges to implementing the programme include:

- imited influence by the medical team on coaching practices
- imited time to include preventive exercises in training before a match
- players indicating that the exercise gives them muscle soreness.

It is also considered unlikely that a single exercise would be the whole solution to a multifactorial injury problem.^27^

### Can we explain why structural injuries were reported to be more common than functional injuries during the recent eight seasons?

We noted some differences in access to imaging and the quality of imaging during the 21 years of this study. In the 2007–2011 period, MRI or ultrasound imaging was obtained in 87% of all hamstring injuries.^28^ In that study, 13% of hamstring injuries showed no MRI signal of abnormality and 57% of injuries had muscle oedema, but no fibre disruption on MRI. These injuries were functional injuries according to the Munich consensus.^9^ The remaining 30% of injuries were radiologically classified as structural.^28^

The 2007–2011 pattern of imaging contrasts starkly with data from the recent years (2011–2018) where 72% of all hamstring injuries were structural, with evidence of muscle tears on MRI. One difference across the two time periods is the quality of MRI scanners. The images reported in 2012 were obtained using 1.0 T and 1.5 T scanners. Since then, many football players have been scanned using 2 T and 3 T magnets which makes the image more likely to appear as Peetrons^29^ grade 2 (structural) than 1 (functional).^29^ There have been considerable hardware and software improvements with MR units, irrespective of field strength. With better coils and resulting improved spatial and contrast resolution, as well as advances in sequence design, even the 1.5 T machines are producing much higher quality images demonstrating more subtle pathology than those of 10 years ago (B Forster, personal communication, 14 October 2022).

### How can our findings be of practical value for players, clinicians and clubs?

In our opinion, collaboration between medical staff, coaches, players and directors will provide the best perspective of how the game of football evolves. Such interdisciplinary discussion is likely to help find solutions to keep players safe and at reduced injury risk.^11^

Our finding that players are exposed to high risk of recurrences during the first 2 months after the index hamstring injury is important for team clinicians. We recommend that clinicians discuss this crucial point with players and coaches/managers/‘the football department’ so that appropriate programmes can be implemented (eg, making all parties aware of the risk, trying to have players complete their rehabilitation diligently, and managing load management in training, and where possible, in matches). We appreciate there is no evidence-based (randomised controlled trial) programme to prevent hamstring recurrence yet.

We found the biceps femoris muscle was injured far more frequently than the semimembranosus/semitendinosus muscles. The reasons for this finding are not well understood, and provide an important focus for future research.

## Methods considerations

A main strength of this cohort study is that its design closely follows the international consensus statements and reporting guidelines for epidemiological research in sport.^12–14^ This allows for our study to be compared with those that followed similar methodologies.^12–14^ Protocol changes are necessary over decades and where this has been appropriate we clearly described what we have done—such as when we began obtaining more detail on the type of hamstring injury and its location.

We took steps to increase the reliability of the collected data, such as a detailed study manual and close communication between the study group and all participating football teams and data collectors.^14^ We note that the set of teams that contribute data to ECIS has differed season by season and this could influence the time-trend analyses. To increase the homogeneity of the cohort over different seasons, the data reported here only include players from teams during seasons in which they qualified for the group stage of the UEFA Champions League.

In this study, we used a time-loss injury definition which means that contact persons were asked to give a date when injured players were considered ready for full return to participation in all team activities. Return to play is often a continuum so the exact date when a player is considered ready to participate fully can be difficult to define. It is also possible that players may return to full participation even though they have persisting symptoms that may affect their ability to perform. These aspects of an injury are not covered using a time-loss definition and are thus not included in our definition of injury burden.

## Data Availability

No data are available. Data sets not available due to confidentiality of players and teams.
